# The Evolution of Intelligence: Analysis of the Journal of Intelligence and Intelligence

**DOI:** 10.3390/jintelligence11020035

**Published:** 2023-02-14

**Authors:** Fabio Andres Parra-Martinez, Ophélie Allyssa Desmet, Jonathan Wai

**Affiliations:** 1 Department of Education Reform, University of Arkansas, Fayetteville, AR 72701, USA; 2 Department of Human Services, Valdosta State University, Valdosta, GA 31698, USA

**Keywords:** bibliometric analysis, scientometrics, intelligence

## Abstract

What are the current trends in intelligence research? This parallel bibliometric analysis covers the two premier journals in the field: Intelligence and the Journal of Intelligence (JOI) between 2013 and 2022. Using Scopus data, this paper extends prior bibliometric articles reporting the evolution of the journal Intelligence from 1977 up to 2018. It includes JOI from its inception, along with Intelligence to the present. Although the journal Intelligence’s growth has declined over time, it remains a stronghold for traditional influential research (average publications per year = 71.2, average citations per article = 17.07, average citations per year = 2.68). JOI shows a steady growth pattern in the number of publications and citations (average publications per year = 33.2, average citations per article = 6.48, total average citations per year = 1.48) since its inception in 2013. Common areas of study across both journals include cognitive ability, fluid intelligence, psychometrics–statistics, *g*-factor, and working memory. Intelligence includes core themes like the Flynn effect, individual differences, and geographic IQ variability. JOI addresses themes such as creativity, personality, and emotional intelligence. We discuss research trends, co-citation networks, thematic maps, and their implications for the future of the two journals and the evolution and future of the scientific study of intelligence.

## 1. Introduction

The scientific study of intelligence has a long and important history of empirical and theoretical contributions. The field of intelligence research has always been multidisciplinary, as cognitive abilities have shown extensive networks of correlations with numerous other phenomena commonly studied across scientific disciplines including psychology, education, cognitive science, and neuroscience (Hunt 2010; Jensen 1998). A large proportion of studies have been published in the journal Intelligence, founded in 1977; however, studies also appear in journals that span the scientific enterprise. Founded in 2013, the Journal of Intelligence (JOI) has become a recognized journal that focuses on the study of intelligence alongside the journal Intelligence. Thus, to uncover the evolution of intelligence research, it is important to examine the research that is published in each of these influential journals to understand not only how intelligence research has evolved to date, but also where it might be going in the future.

This study is an extension of prior bibliometric studies done by Wicherts (2009), Pesta (2018), and Pesta et al. (2018). Wicherts (2009) reported the evolution of research in the journal Intelligence from its inception in 1977 until 2007. Pesta (2018) then updated Wicherts’ work including publications in Intelligence between 2008 and 2015. Subsequently, Pesta et al. (2018) conducted a thematic analysis of article keywords in Intelligence between 2000 and 2016. The first two studies were published in Intelligence, and the latter was published in JOI. Building on these efforts, this paper includes bibliometric analysis of the open access journal JOI since its inception in 2013, as well as the journal Intelligence over the same period. Prior bibliometric work of the journal Intelligence focused on the extent to which keywords were predictive of the number of citations and research influence. In our analysis, we focused on the dynamic nature of the field and examined the relationships among several bibliometric components within the journals, informing the evolution of the field. This study tracks the evolution of how both journals’ contributions have increased and influenced the wealth of knowledge about the scientific study of intelligence in education, psychology, and multidisciplinary fields. Our work combines previous approaches to bibliometric analysis of the journal Intelligence reporting the relative influence of documents published in Intelligence and JOI based on number of citations and author- and journal-level impact metrics. Additionally, we provide a description of the evolution of themes over the past 10 years and current trends in the field using keyword analysis and a discussion of the 10 most-cited documents in each journal.

To understand the publication and thematic trends in the field of intelligence in the last decade, we addressed the following research questions for JOI and Intelligence:

What are the patterns of publications and citations?
Who are the leading and most influential researchers?
What are the most cited papers?
What are the co-citation patterns?
What are the overall thematic trends?
What are the current thematic trends?

## 2. Materials and Methods

Bibliometric analysis is a scientific mapping strategy to identify the primary streams of research over a given time, context, and fields (Van Eck and Waltman 2007). To make a fair comparison, we considered only articles published within the same time frame. Using the database Scopus, we compiled all the publications from both journals between 1 November 2013, and 31 December 2022. This period starts with JOI’s first volume, published in December 2013, and with Intelligence Volume 41, issue 6. We included periodical publications and special issues as both types of publications contribute to the field of intelligence conceptually and empirically. Separate databases were created for each journal. Each database included 31 columns containing information about authors’ names, document titles, keywords, abstracts, type of document, authors’ affiliations, total citations in Scopus, publication date, and reference list. We cleaned the resulting datasets using R code for metatag extraction and citation reference management (Aria and Cuccurullo 2017). This R function segmented each document’s reference list into three additional data columns containing the first author’s last name and year, title, and journal.

Keyword lists often contain synonyms and non-standard words that are used to describe the same topic. To consolidate the trends in keywords, we used Pesta et al.’s (2018) coding book to classify related keywords. Pesta et al. (2018) created umbrella terms or categories to group commonly used keywords and synonyms across similar studies. For example, the term “*g*-factor” was used to categorize other terms like “g”, “general mental ability” and “general cognitive ability.” To correctly classify the number of keywords, we standardized spelling to American English for convenience and reduced plurals to their singular form. For instance, words like “ageing, behaviour, and modelling” were replaced for equivalents “aging, behavior, and modeling”. To classify keywords, we created a mapping algorithm using the Pandas and NumPy libraries on python (Harris et al. 2020). For each publication, the algorithm looked at the list of keywords from each article and compared the keywords with the list of categories and synonyms created by Pesta and colleagues. For each keyword matching a category, the keyword was replaced with the corresponding category. If there was no match after checking all synonyms, the algorithm retained the old keyword. We borrowed 36 pre-established categories and added 11 new categories (see Supplemental Materials). For instance, the new categories included terminology sets such as creativity (creative thinking, innovate, creative achievement, creative aptitude) and artificial intelligence (AI, machine learning, computational modeling, deep learning, brain–computer interface). To prevent the occurrence of duplicates, only one main category was preserved per article. For example, an article containing the keywords *personality-intelligence interface; academic achievement; school performance; latent interaction effect; fluid intelligence; five factor model; personality* was reduced to include only *personality; education; latent interaction effect;* and *fluid intelligence*.

To assess the impact of each journal, we used global and local metrics. We extracted global metrics from three external bibliographic index sources: Scopus, Clarivate, and ScimagoJR (the largest bibliometric databases ranking scientific journals). Common global metrics include journal impact factor, cite score, and *h*-index. Local metrics were derived from the downloaded data. We analyzed the data using the R package Bibliometrix 4.1 (Aria and Cuccurullo 2017; version 13 January 2023). Basic bibliometric elements including number of publications, citation trends, most influential researchers, and keyword frequency were identified in each journal database using the *summary* function and complemented with Scopus metrics. Summary local metrics only account for items in the database. In this case, metrics are calculated on the collection of documents published by each journal and do not reflect author publication metrics outside of JOI and Intelligence.

To assess author influence, we considered the local *h*-index, *g*-index, and *m*-index. The local *h*-index is the number of *h* publications with at least *h* citations within each journal database. For example, an author with an *h*-index of 10 in JOI has at least 10 documents with at least 10 citations in that journal. To assess the *g*-index, all articles in each database were ranked in decreasing order of total citations. Then, we squared the rank of each article. The *g*-index was the largest square rank number, such that the top *g* articles received, together, at least *g*^2^ citations. The *g*-index advantage over the *h*-index is that it gives credit both to documents with high citations (highly influential) while maintaining the contributions of less cited documents (Egghe 2006). The local *m*-index is the authors’ local *h*-index divided by the total number of years an author has actively published in the journal. To examine the most influential articles, we ranked and discussed the most cited documents in both journals.

To establish relationships among authors, references, and keywords, we performed network analysis and visualization of networks to represent relationships using the functions *biblionetwork* and *thematicmap*. These functions allowed us to select the common classification item in the dataset to (a) use the reference column to produce the co-citation network, (b) count the keywords list and produce a co-occurrence network, and (c) combine keywords, authors, and papers to produce a thematic map. Patterns of co-citation inform the extent to which prior research has influenced publications in Intelligence and JOI between 2013 and 2022. To analyze thematic trends, we visualized keyword frequency over time and produced the co-occurrence network for keywords across publications. Co-occurrence networks helped us link keywords commonly used across different papers (Liu et al. 2015). The thematic map function constructs relationships among keywords, papers, and authors to establish the development stage of each topic in the field. We used the VOSviewer application to visualize network data exported from Bibliometrix.

## 3. Results

We retrieved and downloaded bibliometric data for a total of 1101 documents from Intelligence (*n* = 712) and JOI (*n* = 389). Impact metrics, number of publications, citations, authors, keywords, and references position Intelligence as the premier venue for publications in the field between 2013 and 2022. Table 1 shows a summary of each journal’s characteristics according to the database compiled. The impact factor shows the relative influence of a journal based on the number of publications and citations. It is measured by the total number of citations in the current year divided by the number of documents published during the previous two years. For 2022, Intelligence had an impact factor of 3.613, whereas JOI reported 3.176 according to ScimagoJR. Similarly, the Cite Score metric indicates the total number of citations received by a journal in the last four years of publishing activity divided by the number of documents published in that period. For the most recent period assessed in Scopus (2018–2021), Intelligence had a Cite Score of 5.5 and JOI had a Cite Score of 4.

### 3.1. Publication and Citation Trends

**Figure 1:**
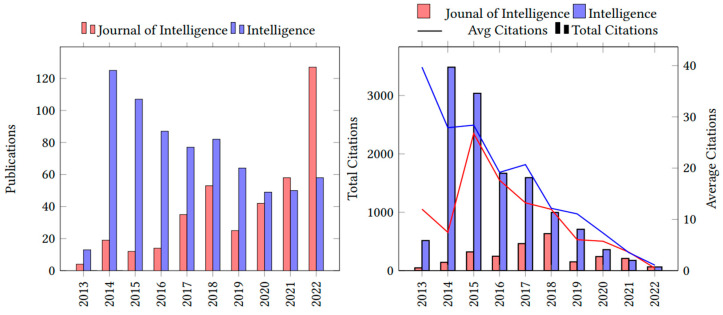
Intelligence and JOI publication and citation growth. The number of citations of an article increases over time. However, the number of citations in the journal as a whole shows a negative tendency, because new papers take longer to be cited than older papers. Avg = average.

Since 2013, Intelligence has published 712 articles (six volumes per year) and attracted 1411 authors. JOI has published 389 articles (four volumes per year) and attracted 878 authors. Figure 1 shows citation and publication trends for the two journals between 2013 and 2022. Overall, Intelligence published the most documents per year (M = 71.2, SD = 31.85), with an average of 17.09 citations per document. JOI shows a steady and accelerated growth pattern in the number of publications, with an average annual growth rate of 46.85%. This growth was influenced by the lower number of publications during the year 2013 (*n* = 4) and the subsequent increase during the following years. These gaps, however, have narrowed over time. Intelligence shows a publication rate of 18.08% over the period of analysis, and this trend is due to a greater number of publications during the years 2014 (*n* = 125) and 2015 (*n* = 107), which then reduced to less than 82 articles each subsequent year. JOI has had, on average, 33.2 publications per year (SD = 22.06) with an average of 6.48 citations per document since its inception in 2013. Following this growth trend, JOI’s number of publications surpassed Intelligence during 2021 and 2022. 

### 3.2. Most Productive and Influential Authors

To list the most productive and influential authors, we ranked each journal’s list of authors using the number of publications, local *g*-index, and *h*-index for all papers published between November 2013 and December 2022. The *g*-index is the largest square rank number such that the top g articles ranked from higher citation to lower citation received together at least *g*^2^ citations. In other words, the *g*-index is the largest perfect square number equal or smaller to the summation of total citations. For example, Robert Sternberg has 156 citations in JOI; ranked in descending order by number of citations, out of 19 articles, his 12th publication squared is equal to 144, which is the perfect square smaller or equal to the total citation count of 156 (12^2^ = 144 < 156). The local *h*-index was calculated separately for each author while considering the h number of publications with at least h citations in each journal. This index is also independent of an author’s global *h*-index based on total publications and citations in other journals throughout their scholarly career. For example, Andreas Demetriou publishes in Intelligence and JOI. Overall, this researcher has a global Scopus *h*-index of 27 (22 *h*-index adjusted for self-citations) for 107 documents and 2502 citations in his entire career. However, in our analysis, Demetriou has a local *h*-index of 9 for 12 publications in Intelligence and a local *h*-index of 4 for six publications in JOI.

Collectively, the top 10 authors in Intelligence account for ~22.5% of total publications and ~24% of total citations. Intelligence attracts influential and productive researchers who have published at least 14 articles in the last 10 years with at least 214 citations. In JOI, the top 10 authors represent 17.7% of total publications and 31.74% of total citations. Table 2 shows a summary of the most productive and influential researchers in the field of intelligence. Intelligence attracts influential and productive researchers who have published at least 11 articles in the last 10 years with at least 214 citations. In this citation-based rank, Ian Deary is the most cited author in Intelligence with a total of 23 articles and 655 (5.2%) citations between 2013 and 2022. For JOI, R. has a total of 19 publications and 156 citations. In this regard, a more accurate measure of an author’s productivity is the fractionalized number of publications, which accounts for the total number of publications divided by the total number of co-authors. In JOI, R. Sternberg has 12.75 fractionalized publications, being the first author in all of them. In Intelligence, the researcher with the highest record of fractionalized publications is Gilles Gignac with 14.75 publications. Self-citation was common for authors in both journals. Self-citation averages varied by author; I. Deary (Intelligence) and R. Sternberg (JOI) used self-citation more frequently than other authors. However, the number of local self-citations did not affect the ranking, as the majority of citations referred to work published before 2013 and outside Intelligence and JOI.

### 3.3. Co-Citation Patterns in JOI and Intelligence

**Figure 2:**
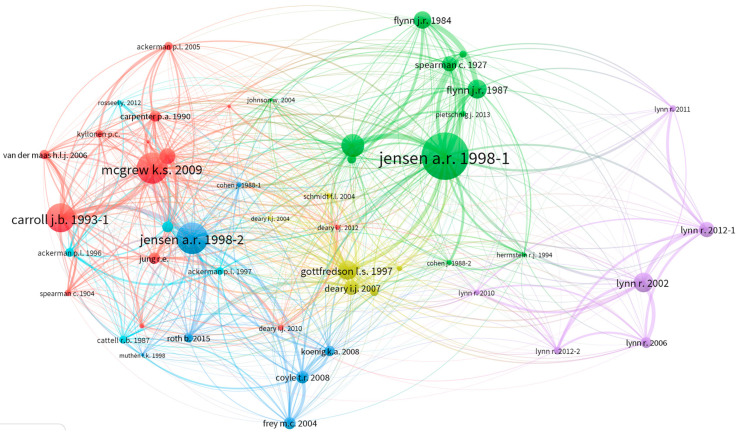
Co-citation network of articles cited in Intelligence. Author names are represented in the format last name and first and/or second initial using Bibliometrix standard lowercase output. For example, jensen a. r. 1998-1 in green indicates publication number 1 in the Scopus database led by Arthur R. Jensen published in 1998. The size of text and node represent relative level of prominence in the network. For example, Jensen (1998) was highly cited between 2013–2022, and often cited together with Flynn (1987) and Spearman (1927).

**Figure 3:**
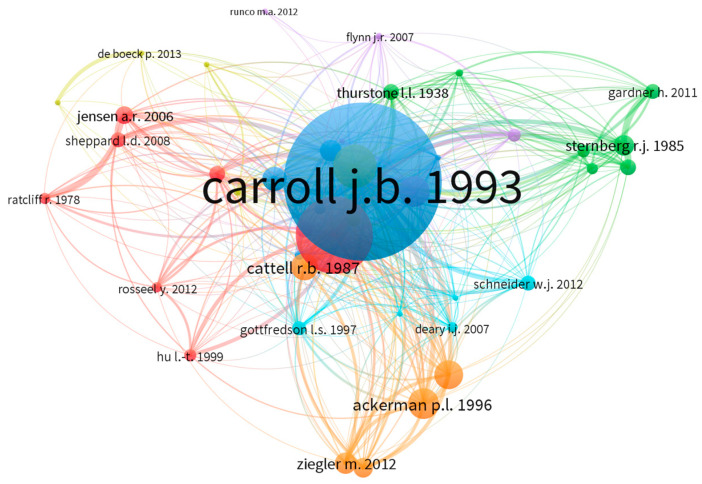
Co-citation network of articles cited in JOI. Author names are represented in the format of last name and first and/or second initial. For example, sternberg r. j. 1985 indicates publication in the Scopus database led by Robert J. Sternberg published in 1985 about the Triarchic Theory of Intelligence, which is commonly co-cited with Gardner’s (2011) theory of Multiple Intelligences. Size of text and node represent relative level of prominence in the network.

Co-citation patterns indicate existing networks articulating conceptual trends and collaborative communities in a field (Boyack and Klavans 2010). In a co-citation network, links are created using an article’s reference list. When two documents are cited together, they are linked with a line, and each document becomes a node in the network. Multiple associated co-citations create clusters that can be interpreted as the body of knowledge influencing current research. Figure 2 and Figure 3 show the co-citation patterns of authors that were cited by papers published in Intelligence and JOI, respectively, between 2013 and 2022. The clusters represent the most common sources that have influenced Intelligence and JOI publications. The co-citations in Intelligence included three main clusters of articles. Cluster one (red) included 14 articles related to models, theories, and components of intelligence. Cluster two (green) included 11 articles with common themes revolving around the Flynn Effect, spearman’s *g*-factor, and the relationship between *g* and cognitive tasks. Cluster three (blue) included five articles by Richard Lynn related to the study of geographic, ethnic, and racial differences in intelligence. The co-citation pattern does not account for author’s self-citation, and the size of the node and proximity reflect how frequently two articles are cited together.

The JOI co-citation network showed six sparsely connected clusters. All the clusters revolved around the foundational work of primarily Carroll (1993) and secondarily Cattell (1987). These co-citations are mainly related to models and theories of intelligence such as the Cattell–Horn–Carroll model of intelligence, Ackerman’s (1996) Theory of Adult Intellectual Development, and Sternberg’s (1985) Triarchic Theory of Intelligence. The green cluster included seven articles focused on the relationship between intelligence and other constructs such as academic achievement, personality, and motivation. The red cluster included articles about memory retrieval, speed of information processing, and the relationships of IQ, skin color, and geographic variables. The blue cluster included co-citations on the relationship between intelligence and education.

### 3.4. Most Cited Works between 2013 and 2022

The number of citations often correlates with an article or a researcher’s popularity and influence in a field (Chan and Grill 2022). We examined the 10 most-cited articles in each journal to understand what types of publications received more attention between 2013 and 2022. Table 3 shows a summary of article author, title, type of article, and citations. A notable difference between the two journals was in the article type. When comparing the two journals, with one exception—a response about criticism on the role of expert performance (Ericsson 2014)—most articles published in Intelligence documented data-driven studies: six empirical studies and two meta-analyses. The articles published in JOI included four conceptual studies, four empirical studies, a review, and a commentary article. Of the twenty articles, seven articles (six in JOI and one in Intelligence) focused on the use of models in intelligence and existing debates on their factorial structure (Beaujean 2015; Cucina and Byle 2017; Eid et al. 2018; Gignac 2016; Morgan et al. 2015; Van Der Maas et al. 2017; van der Maas et al. 2014). Two articles in Intelligence focused on the relationship between creativity and intelligence (Benedek et al. 2014; Karwowski et al. 2016). In JOI, two articles addressed the personality–intelligence relationship and academic achievement (Bergold and Steinmayr 2018; Rammstedt et al. 2018).

Two meta-analyses (Intelligence) and a review (JOI) synthesized research on intelligence. Basten et al. (2015) focused on individual differences and brain function. Roth et al. (2015) investigated relationships between intelligence, school achievement, and potential moderators. Kyllonen and Zu (2016) synthesized existing literature on the role of response time and cognitive ability. In 2014, Intelligence published a special issue on the debate and research about expertise and ability. Two articles from this issue ranked among the most cited articles: Hambrick et al. (2014) critiqued the work of Ericsson et al. (1993) and provided evidence on how deliberate practice is not the only construct explaining expert performance. In the same issue, Ericsson (2014) responded to some of the criticisms of the 1993 article.

Finally, four unrelated articles (three in Intelligence and one in JOI) addressed separate topics of interest to the field. Condon and Revelle (2014) evaluated the psychometric properties of the International Cognitive Ability Resource, an open-access instrument to measure intelligence globally. Von Stumm and Plomin (2015) used latent growth models to assess the IQ gap between low SES and high SES children. De Keersmaecker and Roets (2017) presented experimental results on the relationship between ability level and adjustment of views regarding fake news. Finally, Sternberg (2019) proposed a theory of adaptive intelligence and its role on human survival as a species.

### 3.5. Keyword Analysis and Thematic Trends

Aside from the number of citations, keyword frequency is another popular and informative indicator of the main themes of interest in a research field. Table 4 shows a summary of keyword frequency using Pesta et al.’s (2018) categories. Excluding the general term intelligence, there were similarities in the themes of study shared by the two journals. For example, common top ten keywords in JOI (23.75% of documents) and Intelligence (40% of documents) included *cognitive ability, fluid intelligence, working memory, general intelligence, education*, and *psychometrics.* However, the journals differed in the frequency that these topics were addressed. For example, the top 30 keywords in Intelligence made up 77% of the articles, while JOI top 30 keywords only covered 41.15% of total articles. The first keyword category in JOI was creativity, covering 3.62% of published articles and for Intelligence, intelligence–cognitive ability appeared in 8.6% of articles.

**Figure 4:**
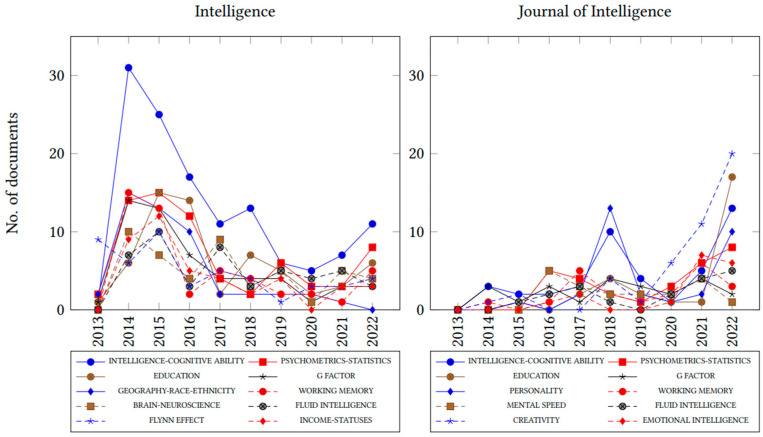
Keyword trends for Intelligence and JOI 2013–2022. Lines and markers represent different keywords in each journal. Only common keywords were represented with the same type of line and marker (intelligence–cognitive ability, education, psychometrics–statistics, working memory, fluid intelligence).

In addition to keyword frequency, trend analysis provides a visual display of how the most popular research themes in the field have evolved over time (see Figure 4). Intelligence had a peak in publications related to cognitive ability, the Flynn effect, psychometrics, and working memory between 2013–2015. However, there was a consistent decline in these major themes during 2019–2020. The study of intelligence related to geography, race, and ethnicity continued to decline through 2022 in both journals. General, crystallized, and fluid intelligence were recurring themes for Intelligence and JOI from 2016 to 2021. The study of personality, creativity, and general intelligence continues to be a trend in JOI, whereas working memory, *g*-factor, and mental speed have plateaued.

We selected 2019–2022 as our preferred period to evaluate the current top 30 keywords in each journal, as such tendencies may inform the direction of the field. To determine current trends in intelligence, we used co-occurrence networks to identify what themes are often published together. This type of network allowed us to build links between articles published during the same period through common keywords. To further examine the role of keywords in research trends, we used thematic mapping to examine the relevance and development of the top 30 keywords used between 2019–2022. A thematic map scores and classifies the co-occurrence network keyword clusters across four quadrants (Aria et al. 2022; Liu et al. 2015). The scores include measures of density and centrality. Centrality (*x*-axis) determines the extent of popularity or relevance of a theme. It is a composite score equivalent to the number of direct connections between the main node and other nodes in the same cluster (betweenness centrality) and the closeness of a node with all other nodes in the network (closeness centrality). For example, a paper keyword connected with multiple keywords directly and indirectly will have a higher degree of centrality, therefore more popularity. Density (*y*-axis) explains the level of development of a topic. It is equivalent to the proportion of actual connections between one node divided by all the potential connections in the network. The upper-left quadrant contains niche topics (low centrality–high density), which are highly specialized areas of research, usually reflecting a small number of publications closely associated by their keywords and high number of citations. The upper-right quadrant contains motor themes for the structure of the research field (high centrality–high density). Motor themes are the core topics that are both highly popular and frequently cited, indicating persistent interest and development in the field. The lower-right quadrant includes basic topics (high centrality–low density) that are highly popular but have not been fully developed or so-called hot topics. Several researchers work on these themes, but they do not accrue many citations. The lower-left quadrant contains emerging or declining topics (low centrality–low density). Usually, they are less cited and less popular topics. In the network, these topics are relatively peripheral and could be considered emerging topics if they are novel, or declining topics, indicating the field is moving in other directions.

**Figure 5:**
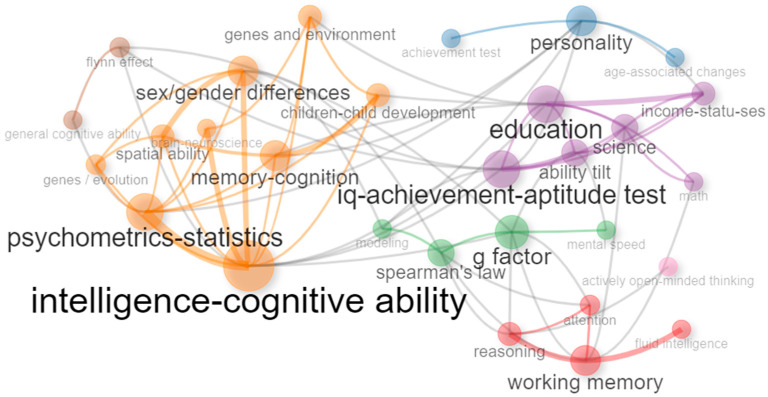
Intelligence journal keyword network 2019–2022. This network represents the top 30 keywords used across 46 out of 58 articles published between 2019 and 2022. Node size is relative to the number of publications containing a keyword. Link thickness indicates the frequency of two words occurring together in more than one publication.

Figure 5 shows the co-occurrence networks. Intelligence has a cohesive network with six interconnected clusters. A link is built between two different articles using the same keyword. The size of nodes is relative to the frequency of the keywords in the cluster. Central clusters stemming from intelligence–cognitive ability (nine nodes, frequency 11 articles), education (six nodes, six articles), working memory (four nodes, five articles), *g*-factor (four nodes, thre articles). Peripheral themes included personality (three nodes, three articles) and the Flynn effect (two nodes, four articles).

**Figure 6:**
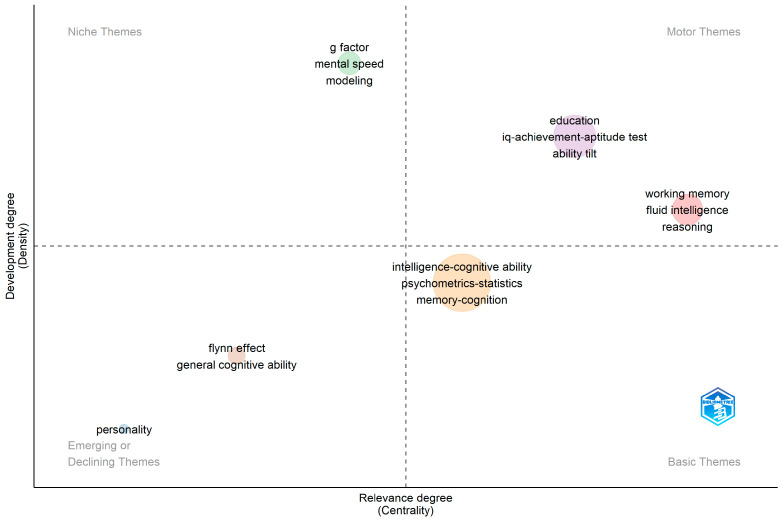
Keyword thematic map for the journal Intelligence. Only three keywords are displayed per cluster for clarity of visualization. Size of cluster is relative to the number of documents. Only documents published during 2022 are displayed in the plot. Motor or core themes indicate high development and importance for the field. Niche themes (specialized) are less popular but highly developed. Basic themes are common important areas of research with low relative development, e.g., hot topics. Emerging or declining themes have low relative importance and development.

Figure 6 shows the corresponding thematic map for the previous network, indicating the relevance of research themes. The map represents 58 articles grouped in six clusters. Core topics comprised the two clusters of education and working memory. The education cluster with words such as IQ–achievement–aptitude tests and ability tilt included seven articles (see Cave et al. 2022; Coyle 2022a; Wai et al. 2022; Zisman and Ganzach 2022). Working memory included seven articles and was connected with fluid intelligence, reasoning, and attention (Burgoyne et al. 2022; Demetriou et al. 2022; Erceg et al. 2022; Tourva and Spanoudis 2020). The keyword intelligence–cognitive ability had the most direct connections with other nodes and is one of the basic clusters in research in Intelligence. However, it ranked low in centrality because the direct nodes were not highly associated with other nodes. Therefore, the centrality of the node is high, but the centrality of the cluster is averaged down. Basic themes included psychometrics and statistics, memory–cognition, and sex–gender differences (see Coyle 2022b, Geary 2022; Laureys et al. 2022; Otero et al. 2022). A highly niche theme included *g*-factor, mental speed, and Spearman’s Law (see Coyle 2022a; Feraco and Cona 2022; Tatel et al. 2022). Peripheral declining themes included the Flynn effect and general cognitive ability (see Gonthier and Grégoire 2022) and personality (see Ganzach and Zisman 2022; Rusche and Ziegler 2022).

**Figure 7:**
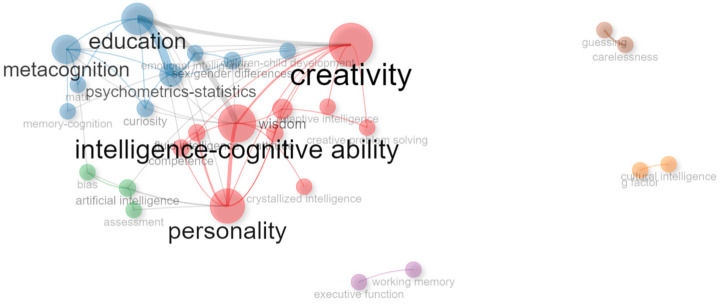
JOI keyword network 2019–2022. This network represents the top 30 keywords used across 86 out of 127 articles published in JOI between 2019 and 2022. Node size is relative to the number of publications containing a keyword. Link thickness indicates the frequency of two words occurring together in more than one publication.

JOI’s keyword network had three cohesive central clusters and three isolated clusters (Figure 7). Creativity, personality, and intelligence-cognitive ability formed the largest cluster (10 nodes, 34 articles); followed by education and metacognition (9 nodes, 35 articles); and artificial intelligence (3 nodes, 8 articles). These three clusters showed high frequency in the number of articles using the keyword and the number of links between nodes. Additionally, there were multiple links between education and creativity, and education and intelligence-cognitive ability. The three isolated peripheral clusters included working memory (2 nodes, 4 articles), cultural intelligence (2 nodes, 3 articles) and item guessing (2 nodes, 2 articles).

**Figure 8:**
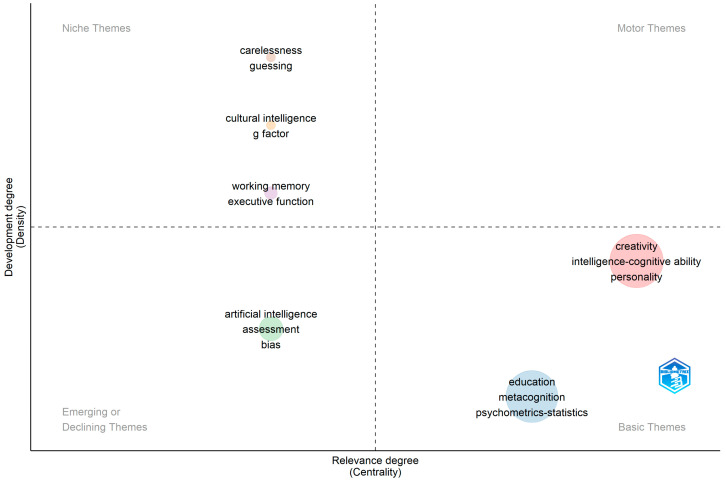
Keyword thematic map for JOI. Only three keywords are displayed per cluster for clarity of visualization. Size of cluster is relative to the number of documents. Only documents published during 2022 are displayed in the plot. Motor or core themes indicate high development and importance for the field. Niche themes (specialized) are less popular but highly developed. Basic themes are common important areas of research with low relative development, e.g., hot topics. Emerging or declining themes have low relative importance and development.

The thematic map for JOI showed a relatively high level of centrality for the creativity and education clusters. However, these clusters were only found in the basic quadrant, indicating high popularity but low level of development of topics using the keywords creativity, intelligence, personality, and wisdom (see Beghetto and Madison 2022; Childs et al. 2022; Massie et al. 2022; Suh and Ahn 2022) and education, metacognition, psychometrics-statistics, and emotional intelligence (see Forthmann et al. 2022; Hofer et al. 2022; Józsa et al. 2022; Novikova et al. 2022). An emerging topic was artificial intelligence blended with bias and assessment (see Andrews-Todd et al. 2022; Bernardo et al. 2022; Pásztor et al. 2022). No articles were ranked in the core (motor) topic’s quadrant. This journal also showed three separate niched clusters indicating only few researchers used the keywords working memory and executive function (see Panesi et al. 2022; Rosas et al. 2022) cultural intelligence and the *g*-factor (Alifuddin and Widodo 2022; Sternberg 2022; Sternberg et al. 2022), and items carelessness and guessing (Antoniou et al. 2022; Sideridis and Alahmadi 2022). Figure 8 shows the thematic map of keywords in JOI.

## 4. Discussion

By analyzing data such as publication counts, citation counts, and co-authorship networks, bibliometric analysis can help researchers and institutions to identify key players and influential publications, as well as areas in which more research is needed. This bibliometric analysis informs the evolution of the field of intelligence over the past decade as established by trends in the most important publication venues. We expanded the work of Wicherts (2009) and Pesta (2018), published in Intelligence, and Pesta et al. (2018), published in JOI. We added the comparison in JOI after its introduction to the field and implemented network analysis and thematic mapping to dissect the relationships among researchers, publications, citations, and topic trends.

### 4.1. Journal Reputation and Growth

Studies by Wicherts (2009) and Pesta (2018) focused on the most influential authors and articles based on citation number and rank. Wicherts’ analysis evaluated the extent to which articles published in Intelligence attracted high citation counts and found that only 3.1% of published articles were not cited at the time of his analysis. For Wicherts, a reduced number of uncited documents was a sign of the high reputation of Intelligence in the field. To date, only 8.5% of articles published in Intelligence have not been cited. This relative increase in the number of uncited articles can be attributed to three potential causes: (a) more recent publications need more time to be cited, (b) the explosion of publications during the last decade makes it more difficult for articles to reach an audience to be considered for citation, and (c) the inception of JOI as a venue for intelligence research gives the research audience more options to cite articles. Being an open-access journal, JOI has the advantage in attracting readership as it lacks the obstacle of paywalls. While JOI has been active for only a decade, the journal has rapidly attracted influential authors and has enjoyed a positive reception from the field. Comparatively, JOI has surpassed the number of Intelligence publications per year since 2021 and continues to grow at 2.69 times the growth rate of Intelligence. However, this growth has not been matched by the number of citations received by JOI. The volume of uncited documents composes about 30% of total publications. The performance of both journals on average citations per article per year remains relatively close at present. Based on bibliographic databases, JOI’s impact has grown steadily, as reflected in the journal impact factor and cite scores across Clarivate Analytics, Scopus, and ScimagoJR. Therefore, we expect that the growth will continue over the years to come, but confirmation will require further study.

### 4.2. Productive and Influential Researchers

Several of the authors featured in Wicherts’ and Pesta’s separate analyses continue to make noteworthy contributions to the field. Our analysis indicated that Ian Deary, Roberto Colom, Richard Lynn, Jan te Nijenhuis, and Andreas Demetriou continue to rank among the top 10 authors publishing in Intelligence. JOI has attracted multiple publications from researchers such as Robert Sternberg, Han van der Maas, Andrew Conway, Samuel Greiff, and Oliver Wilhelm. It is important to highlight that while the *h*-index is an indicator of influence, it fails to explain an author’s general productivity and impact, as it does not account for collaborations with multiple authors or the extent to which each author contributed to a publication. For both journals, we faced the challenge of comprehensively accounting for author collaborations. For example, Demetriou and Spanoudis shared five papers together. Using traditional metrics, both researchers would receive the same amount of credit based on their publications and citations. Another challenge is the use of self-citation. To adjust for the influence of self-citation, we used the adjusted *h*-index, which removes all self-citations from an author’s global *h*-index score. Nevertheless, self-citation was common in both journals, with top first authors Ian Deary losing 8 *h*-points and Robert Sternberg 6 *h*-points. Overall, Intelligence’s top authors had their *h*-index reduced between 2 and 8 points, while JOI authors lost between 2 and 6 points.

While author productivity reputation matters for journals, the top-ten list lacked gender, geographic, and racial representation. Both journals’ rankings were male-dominated, with JOI including only one female author, Anna-Lena Schubert. Intelligence did not have female authors in its list. Most researchers represented North America and Europe (only 10 countries accounted for nearly 60% of articles with the 1st corresponding author). Perhaps this is an opportunity for the intelligence research field to encourage and support more diverse researchers on numerous dimensions to publish in these journals and join the international community of researchers that study intelligence.

### 4.3. Most Cited Papers

Identifying the most cited papers is a traditional practice in bibliometric studies. It helps to outline the topics and methodological strategies that influence a field. Wicherts (2009) identified the most important papers and topics between 1970–2009. His work highlighted topics such as working memory (Conway et al. 2002; Kyllonen and Christal 1990), the debate of the factor structure of *g* (Gustafsson 1984), emotional intelligence (Mayer et al. 1999; Mayer and Salovey 1993), child development and intelligence (Fagan and McGrath 1981), the relationship between brain functioning–size and intelligence (Haier et al. 1988; Willerman et al. 1991), and individual differences (Deary et al. 2000). Pesta (2018) identified similar trends between 2009 and 2017 (see Colom et al. 2008; Johnson et al. 2008; Karama et al. 2009; McGrew 2009; Oberauer et al. 2008). From that period, there was also research on geographical variations in IQ (Lynn et al. 2009; Lynn and Meisenberg 2010), the relationship between creativity and intelligence (Jauk et al. 2013; Nusbaum and Silvia 2011), and achievement tests and intelligence (Deary et al. 2007; Koenig et al. 2008). Our findings reflect persistent trends in these topics among the most cited articles in Intelligence. One difference, perhaps, is the inclusion of large-scale assessments as well as novel statistical techniques contributing to better model estimates to measure intelligence. For example, von Stumm and Plomin (2015) provided evidence of SES gaps using a longitudinal sample of twins including 14,853 children. Gignac (2016) used 12 simulated matrices to test the proportionality hypothesis for a higher-order factor versus the bi-factor model of intelligence. The wealth of intelligence research accumulated over the last 40 years has also allowed for synthesis of the research corpus. A meta-analysis supported the positive relationship between intelligence and school performance (Roth et al. 2015). Another meta-analysis focused on the biological basis of intelligence through the study of brain images; these authors argued, among other things, that the frontal and parietal brain regions are important for human intelligence (Basten et al. 2015). The relationship between creativity and intelligence is another classic topic that makes it to the most-cited paper list. The most cited article in Intelligence (Benedek et al. 2014) argued that the relationship between creativity and intelligence can be explained by executive abilities such as updating, shifting, and inhibition. In the same journal, Karwowski et al. (2016) conducted eight studies to test the relationship between intelligence and creativity, arguing that intelligence is necessary but not sufficient to explain creative thinking.

Interestingly, while new in the field, JOI had its own share of all-time classic themes. The most cited articles in JOI investigated the factor structure of intelligence. Using Monte Carlo simulations to test the relationship between fit indices and bi-factor, multifactor, and hierarchical models of intelligence, Morgan et al. (2015) suggested the need for conceptually and theoretically driven interpretations of models, rather than just following data-driven interpretations. In 2014, a commentary article on the mutualism models as an alternative to latent models applied an index scoring structure and the role of environmental variables to measure and explain intelligence (van der Maas et al. 2014). A conceptual article discussed how using mathematical and mechanistic network models could potentially reconcile the divide between cognition and intelligence research (Van Der Maas et al. 2017). Specifically, the researchers proposed “a new unified network model of general intelligence that incorporates four basic explanations: mutualism between basic cognitive processes during development, multiplier effects through the environment, sampling in manifest test scores, and centrality of key processes such as working memory.” (p. 13). Beaujean’s (2015) conceptual article argued for the bi-factor model as John Caroll’s true view on intelligence. Then, in 2017, Cucina and Byle (2017) found evidence supporting the bi-factor model using an historical archive of 58 datasets and 1.7 million test-takers. Research for and against the factor structures of intelligence highlights once more that the field of intelligence continues to hold different viewpoints, and unified understandings and shared models may allow us to strengthen our understanding. Additionally, some scholars attest that a strong empirical argument in favor of one model over the other is also lacking. Researchers have reported at times conflicting findings, with several demonstrating through simulations that the bi-factor model results in a marginally better model fit than the higher-order model (Cucina and Byle 2017; Eid et al. 2018). However, model fit differences were, in many cases, negligible and did not change conclusions in absolute terms. For those reasons, as well as the fact that the relative statistical and practical advantages of each model may be context-dependent, Carroll (1993) continues to be highly influential.

While researchers in Intelligence addressed creativity, two studies in JOI addressed the role of personality. Bergold and Steinmayr (2018) investigated the moderator effect of personality traits on the relationship between intelligence and academic achievement on two samples of 11th graders, concluding that achievement was highly correlated with intelligence when levels of conscientiousness were also high. Another study introduced a novel facet-level application to test the relationship between components of intelligence and personality (Rammstedt et al. 2018). The researchers concluded that the relationship is in fact nuanced, and global models may fail to depict the relationship accurately.

Two articles in Intelligence and one article in JOI broke with traditional topics and attempted to connect intelligence research with current topics. Condon and Revelle’s (2014) paper documented the reliability and validity of a public-domain measure of cognitive ability and established precedence for using public-domain measures in the field. De Keersmaecker and Roets (2017) studied the role of cognitive ability on the impact of false information. They found that the degree to which people correct their judgments depends on their cognitive ability. In JOI, Sternberg (2019) argued for a Theory of Adaptive Intelligence, emphasizing intelligence’s role for the common good and human collective survival as a species.

Finally, all empirical papers used only quantitative methods. Most studies had a female-dominated sample (anywhere from 50.3% to 77.7% female). These two findings suggest an opportunity for diversifying samples for the study of intelligence, which may be addressed in part by implementing inclusive and multiple methodological perspectives. Regarding participant sampling, the field could also improve in the inclusion of underrepresented populations as in psychology and education research (Cole 2009; Rad et al. 2018).

### 4.4. Thematic Trends

One of the main contributions of our work consists of standardizing and mapping keywords on cumulative and relative frequency to identify the trends and directions of the field. Pesta and colleagues (2018) used keyword analysis and focused on the association of keywords, number of citations, and topic frequency over time on all articles published in Intelligence between 2000 and 2017. Pesta concluded that keyword choice did not correlate with the number of citations. Building upon their work, we expanded their original codebook of common keywords and categories. We created an algorithm to map keywords in papers to pre-established categories. This allows for easy and automatic replacement and classification of themes. In Pesta et al. (2018) the top 10 keywords included *g*-factor, psychometrics–statistics, education, IQ–achievement–aptitude test, race–ethnicity, working memory, brain–neuroscience, nature–nurture, and children–child development. Regarding overlap in our findings between 2013 and 2017, only one category differed between the two studies. In our sample, the Flynn Effect replaced nature–nurture. This keyword category was found in 52 papers. The top 10 categories in Intelligence accounted for 42.64% of keywords. JOI top 10 keywords included creativity, personality, emotional intelligence, and mental speed, accounting for 9.57% of all keywords. The large discrepancy in the use of keywords and their varying proportions between the two journals suggests that JOI overall attracts researchers with more variation in research agendas, and therefore, includes more diverse topics according to keyword frequency. This finding was confirmed with the change of the top 10 keywords over time. In JOI, creativity, personality, education, and emotional intelligence have grown in use since 2019. Intelligence’s growth in keyword use centers around intelligence–cognitive ability and psychometrics–statistics. Two declining trends include fluid intelligence and geography–race–ethnicity. Another potential explanation for the discrepancy between journals could be that Intelligence may have standardized keywords or words that are unique to its contributors, whereas JOI may not necessarily have a systematic record of keywords. This speculation requires further study. A strategy to effectively address this issue involves unification of terminology between the two journals to reduce confusion and incorporate nuanced contextual meanings of keywords.

JOI and Intelligence keyword networks and thematic maps in 2022 point to the directions the field is currently taking. The most popular and frequently developed topics in Intelligence are related to cognitive ability, working memory, *g*-factor, and education. A novel trend is marked by publications studying the role of personality. These thematic trends suggest that Intelligence may be a venue that has a well-established tradition, with core topics, basic, and niche themes focused on well-defined historical and foundational boundaries—hence the few node connections and relationships across topic clusters. Nonetheless, Intelligence has made efforts to promote the discussion of the future of the field in the light of trends such as advances in AI, genetics, and neuroscience. Intelligence published a special issue in 2021 devoted to the future of intelligence research addressing critical perspectives on definitions, models, measures, and the history of intelligence research (see Coyle and Greiff 2021; Demetriou et al. 2021; Euler and Schubert 2021; Haier 2021; Koch et al. 2021; Neubauer 2021; van der Maas et al. 2021; Wai and Worrell 2021; Wilhelm and Kyllonen 2021).

Compared to Intelligence, JOI seems to have moved away from debates and research on race, ethnicity, and geographic differences as well as becoming less focused on traditional models of intelligence. Moreover, JOI has focused on current “hot” topics on other science areas including creativity, emotional intelligence, and personality that were highly interconnected among clusters during 2022. JOI has included discussion of the importance of considering the most effective ways to communicate intelligence research given ongoing historical challenges to the field (Wai 2020), and Intelligence has included discussion on how fields that are focused largely on empirical science such as intelligence research may not be easily integrated with more applied fields influenced by politics and values, such as education (Wai and Worrell 2021). However, while JOI is open to novelty and popular themes, it lacks a set of well-developed topics that serve as a core for the journal. One area that favored JOI in recent publications was the inclusion of novel themes such as artificial intelligence and machine learning to address old problems in the field such as the need for psychometrically sound instruments and reduction of bias or by combining current topics such as creativity with novel tendencies (Bernardo et al. 2022; Marrone et al. 2022). JOI might leverage its potential as an open-access journal to reach greater audiences and influence not only the field of intelligence, but also expand through other multidisciplinary avenues. At the same time, better ensuring that hot topics are integrated and empirically tested against widely established historical findings remains critical. Some of the challenges to the field, and perhaps some directions to JOI, were hinted in the journal’s opening editorial article by Hunt and Jaeggi (2013). A special issue addressing new directions in the light of the findings presented in this study may be potentially useful.

## 5. Limitations and Conclusions

This bibliometric study provides valuable insights into the trends and challenges of intelligence research. We identified key topics, authors, and journals that are driving the field forward and identified areas where further research is needed. While carefully crafted, this study has methodological limitations. Bibliometric analysis was restricted to the availability of metadata and bibliometric information. By using the Scopus database, our results may differ from other bibliometric databases such as the Web of Science, Google scholar, or PubMed. We included all documents published between 2013 and 2022, as they contributed to the number of publications and citations. A challenge of this inclusive approach is that results cannot be discriminated by the types of publications (e.g., letters, editorials, rebuttals, etc.). Additionally, by including special issues, it is possible that the peer-review process might not be equivalent for special articles than for regular publications (JOI in particular has numerous special issues). While we built upon prior bibliometric studies to build a dictionary of categories and synonyms for the keywords, more work is necessary to develop a comprehensive and accurate repository of common keywords that are useful to disambiguate confusion among authors and readers. An important limitation of this analysis, which regards the discussion of thematic issues and trends, is that the most cited and popular papers are not necessarily about the most empirically supported constructs. The replication crisis in psychology is an excellent illustration of how novel topics can be exciting but may not necessarily hold up over time. Thus, focusing on the past decade of intelligence research in two major journals is useful to track recent topics and trends, but may not necessarily reflect what ideas actually survive the test of time based on the broader body of evidence. At the same time, our paper illustrates the most exciting new topics in the field of intelligence research in two overlapping yet distinct communities of intelligence researchers as reflected in the journals Intelligence and JOI. The evolution and future of intelligence research is important to track, and bibliometric analyses may be useful to help understand both the past, present, and future of the scientific study of intelligence and the scholars who compose the community of intelligence researchers around the world.

## Figures and Tables

**Table 1:** Summary of Intelligence and JOI publication bibliometric data.

Description	Intelligence	JOI
Journal Metrics		
Impact Factor (Clarivate Analytics)	3.613	3.176
Cite Score (Scopus)	5.5	4.0
Global *h*-index (ScimagoJR)	98	18
Local *h*-index (since 2013)	49	22
Local *g*-index	74	34
Local *m*-index	4.45	2
Total Citations	12,605	2520
Core information about data 2013–2022		
Documents	712	389
Annual growth rate	18.08	46.85 ^1^
Document average age	5.83 years	3.47 years
Average citations per document	17.07	6.48
Average citations per year per document	2.68	1.48
References	34,729	24,285
Authors keywords	1548	1327
Authors		
Total authors	1411	878
Unique authors of single-authored documents	79	61
International co-authorships	37.78%	27.60%
Author Collaboration		
Single-authored documents	132	85
Documents per author	0.50	0.44
Co-authors per document	3.34	2.81
Document Types		
Article	641	322
Editorial	10	13
Letter	3	3
Note/errata	27	27
Review	31	24

^1^ For the year 2013, starting on November 1st, only 4 documents were published in JOI and 13 in Intelligence. This is the baseline to calculate publication growth.

**Table 2:** Most influential and productive authors.

Journal	Local *g*-Index *	Author	Total Papers	First Author	Fraction Papers	Local *h*-Index	Local *m*-Index	Global *h*-Index **	Adjusted *h*-Index Self-Cite **	Total Citations	Average Citations	Average Self Cites	Percent Journal Cites
INT	23	Deary IJ.	23	4	6.78	13	1.3	152	144	655	28.48	13.09	5.2%
INT	23	Gignac G.	23	18	14.75	13	1.3	31	29	551	23.96	5.09	4.4%
INT	20	Te Nijenhuis J.	20	10	5.38	12	1.091	23	21	432	21.60	5.05	3.4%
INT	20	Lynn R.	24	7	9.77	11	1	40	38	429	17.88	8.42	3.4%
INT	16	Greiff S.	16	1	4.53	8	1	31	26	274	17.13	9.13	2.2%
INT	14	Wilhelm O.	14	1	4.05	10	1	38	35	257	18.36	7.43	2.0%
INT	13	Coyle TR.	15	13	10.12	9	0.9	23	19	191	12.73	6.33	1.5%
INT	13	Colom R.	13	0	4.05	7	0.7	44	41	182	14.00	6.77	1.4%
INT	12	Demetriou A.	12	7	3.44	9	0.9	27	22	277	23.08	10.50	2.2%
INT	11	Spanoudis G.	11	0	3.77	8	0.8	22	18	214	19.45	8.18	1.7%
JOI	12	Sternberg RJ.	19	19	12.75	8	0.8	94	88	156	8.21	15.95	6.2%
JOI	9	Wilhelm O.	9	2	3.13	5	0.625	38	35	91	10.11	10.67	3.6%
JOI	8	Schubert AL.	8	3	2.40	6	0.75	15	14	127	15.88	3.38	5.0%
JOI	6	Forthmann B.	6	3	1.73	3	0.75	14	11	46	7.67	2.50	1.8%
JOI	6	Greiff S.	6	2	1.87	3	0.333	31	26	48	8.00	1.67	1.9%
JOI	6	Demetriou A.	6	5	1.94	4	0.571	27	22	57	9.50	9.67	2.3%
JOI	6	Schmitz F.	6	3	2.04	5	0.625	19	18	76	12.67	7.17	3.0%
JOI	6	Ziegler M.	6	3	2.65	4	0.4	31	28	82	13.67	8.00	3.3%
JOI	6	Frischkorn GT.	6	2	1.95	5	0.625	10	9	89	14.83	5.00	3.5%
JOI	6	Van der Maas HLJ.	7	2	1.40	4	0.4	46	45	171	24.43	3.86	6.8%

* Articles are ranked based on the *g*-index = top g articles with g citations in the database. ** Global and adjusted *h*-indexes are used for comparison of authors’ influence outside Intelligence and JOI. INT = the journal Intelligence.

**Table 3:** Most cited articles in Intelligence and JOI between 2013 and 2022. Citation count taken on 13 January 2023 through Scopus.

Article	Citations	Type	Article	Citations	Type
Benedek et al. 2014. Intelligence creativity and cognitive control	372	Empirical	Morgan et al. 2015. Are fit indices biased in favor of bi-factor models	109	Empirical
Roth et al. 2015. Intelligence and school grades. Meta-analysis	255	Meta-analysis	Van Der Maas et al. 2017. Network models for cognitive development	78	Theoretical Conceptual
Hambrick et al. 2014. Deliberate Practice. Is that all it takes to become an expert?	203	Empirical	Kyllonen and Zu. 2016. Use of response time for measuring cognitive ability	59	Review
Condon and Revelle. 2014. The international cognitive ability resource	178	Empirical	Beaujean. 2015. John Carroll’s views on intelligence	54	Theoretical Conceptual
De Keersmaecker and Roets. 2017. Fake news incorrect but hard to correct	159	Empirical	Cucina and Byle. 2017. The bifactor model fits better than higher order models	54	Empirical
Basten et al. 2015. Where smart brains are different. Meta-analysis	157	Meta-analysis	Van Der Maas et al. 2014. Intelligence is what intelligence tests measure.	50	Comment
von Stumm and Plomin. 2015. Socioeconomic status and the growth of intelligence	145	Empirical	Bergold and Steinmayr. 2018. Personality and intelligence interact to predict academic achievement.	41	Empirical
Karwowski et al. 2016. Is creativity without intelligence possible?	136	Empirical	Eid et al. 2018. Bifactor models for predicting criteria by general and specific factors.	38	Empirical
Gignac. 2016. The higher-order model imposes a proportionality constraint	123	Empirical	Sternberg. 2019. A theory of adaptive intelligence and its relation to general intelligence	37	Theoretical conceptual
Ericsson. 2014. Why expert performance is special and cannot be extrapolated.	117	Response	Rammstedt et al. 2018. Relationships between personality and cognitive ability: a facet-level analysis	37	Empirical

**Table 4:** Keyword frequency for Intelligence and JOI.

	Intelligence				JOI		
Rank	Words	Count	Percent	Rank	Words	Count	Percent
*	Intelligence	255	16.47%	*	Intelligence	113	8.52%
1	Intelligence–Cognitive Ability	133	8.59%	1	Creativity	48	3.62%
2	Psychometrics–Statistics	80	5.17%	2	Intelligence–Cognitive Ability	45	3.39%
3	Education	66	4.26%	3	Personality	39	2.94%
4	Geography–Race–Ethnicity	61	3.94%	4	Education	34	2.56%
5	Children–Child Development	58	3.75%	5	Psychometrics–Statistics	33	2.49%
6	Brain–Neuroscience	56	3.62%	6	Children–Child Development	31	2.34%
7	*g* Factor	56	3.62%	7	*g* Factor	24	1.81%
8	Flynn Effect	52	3.36%	8	Working Memory	21	1.58%
9	IQ–Achievement–Aptitude Test	49	3.17%	9	Emotional Intelligence	20	1.51%
10	Working Memory	49	3.17%	10	Mental Speed	20	1.51%
11	Fluid Intelligence	48	3.10%	11	IQ–Achievement–Aptitude Test	19	1.43%
12	Income–Status–SES	48	3.10%	12	Fluid Intelligence	18	1.36%
13	Memory–Cognition	39	2.52%	13	Individual Differences	15	1.13%
14	Sex/Gender Differences	35	2.26%	14	Reasoning	15	1.13%
15	Genes/Evolution	34	2.20%	15	Memory–Cognition	14	1.06%
16	Adult–Aging	30	1.94%	16	Modeling	14	1.06%
17	Crystallized Intelligence	29	1.87%	17	Complex Problem Solving	13	0.98%
18	Health	29	1.87%	18	Attention	12	0.90%
19	Personality	29	1.87%	19	Adult–Aging	11	0.83%
20	Creativity	27	1.74%	20	Executive Function	10	0.75%
21	Modeling	24	1.55%	21	Genes/Evolution	10	0.75%
22	Elementary Cognitive Task	23	1.49%	22	Wisdom	10	0.75%
23	Mental Speed	22	1.42%	23	Assessment	9	0.68%
24	Raven’s	19	1.23%	24	Brain–Neuroscience	9	0.68%
25	Expertise	18	1.16%	25	Elementary Cognitive Task	9	0.68%
26	Genes and Environment	18	1.16%	26	Flynn Effect	9	0.68%
27	Longitudinal	16	1.03%	27	Longitudinal	9	0.68%
28	Ability Tilt	15	0.97%	28	Metacognition	9	0.68%
29	Politics	15	0.97%	29	Crystallized Intelligence	8	0.60%
30	Artificial Intelligence	14	0.90%	30	Factor Analysis	8	0.60%
	Cumulative	1192	77%		Cumulative	546	41.15%

## Data Availability

Data are contained within the article or supplementary material.

## Supplementary Materials

The following supporting information can be downloaded at https://www.mdpi.com/article/10.3390/jintelligence11020035/s1.

## References

[B1-jintelligence-11-00035] Ackerman Philip. L. (1996). A Theory of Adult Intellectual Development: Process, Personality, Interests, and Knowledge. Intelligence.

[B2-jintelligence-11-00035] Alifuddin Moh, Widodo Widodo (2022). How Is Cultural Intelligence Related to Human Behavior?. Journal of Intelligence.

[B3-jintelligence-11-00035] Andrews-Todd Jessica, Steinberg Jonathan, Flor Michael, Forsyth Carol M. (2022). Exploring Automated Classification Approaches to Advance the Assessment of Collaborative Problem Solving Skills. Journal of Intelligence.

[B4-jintelligence-11-00035] Antoniou Faye, Alkhadim Ghadah, Mouzaki Angeliki, Simos Ppanagiotis (2022). A Psychometric Analysis of Raven’s Colored Progressive Matrices: Evaluating Guessing and Carelessness Using the 4PL Item Response Theory Model. Journal of Intelligence.

[B5-jintelligence-11-00035] Aria Massimo, Cuccurullo Corrado (2017). Bibliometrix: An R-tool for Comprehensive Science Mapping Analysis. Journal of Informetrics.

[B6-jintelligence-11-00035] Aria Massimo, Cuccurullo Corrado, D’Aniello Luca, Misuraca Michelangelo, Spano Maria (2022). Thematic Analysis as a New Culturomic Tool: The Social Media Coverage on COVID-19 Pandemic in Italy. Sustainability.

[B7-jintelligence-11-00035] Basten Ulrike, Hilger Kirsten, Fiebach Christian J. (2015). Where smart brains are different: A quantitative meta-analysis of functional and structural brain imaging studies on intelligence. Intelligence.

[B8-jintelligence-11-00035] Beaujean Alexander (2015). John Carroll’s Views on Intelligence: Bi-Factor vs. Higher-Order Models. Journal of Intelligence.

[B9-jintelligence-11-00035] Beghetto Ronald A., Madison Ed (2022). Accepting the Challenge: Helping Schools Get Smarter about Supporting Students’ Creative Collaboration and Communication in a Changing World. Journal of Intelligence.

[B10-jintelligence-11-00035] Benedek Mathias, Jauk Emanuel, Sommer Markus, Arendasy Martin, Neubauer Aljoscha C. (2014). Intelligence, creativity, and cognitive control: The common and differential involvement of executive functions in intelligence and creativity. Intelligence.

[B11-jintelligence-11-00035] Bergold Sebastian, Steinmayr Ricarda (2018). Personality and intelligence interact in the prediction of academic achievement. Journal of Intelligence.

[B12-jintelligence-11-00035] Bernardo Allan B. I., Cordel Macario O., Lapinid Minie Rose C., Teves Jude Michael M., Yap Sashmir A., Chua Unisse C. (2022). Contrasting Profiles of Low-Performing Mathematics Students in Public and Private Schools in the Philippines: Insights from Machine Learning. Journal of Intelligence.

[B13-jintelligence-11-00035] Boyack Kevin W., Klavans Richard (2010). Co-citation Analysis, Bibliographic Coupling, and Direct Citation: Which Citation Approach Represents the Research Front Most Accurately?. Journal of the American Society for Information Science and Technology.

[B14-jintelligence-11-00035] Burgoyne Alex P., Mashburn Cody A., Tsukahara Jason S., Engle Randall W. (2022). Attention control and process overlap theory: Searching for cognitive processes underpinning the positive manifold. Intelligence.

[B15-jintelligence-11-00035] Carroll John B. (1993). Human Cognitive Abilities: A Survey of Factor-Analytic Studies.

[B16-jintelligence-11-00035] Cattell Raymond B. (1987). Intelligence: Its Structure, Growth and Action.

[B17-jintelligence-11-00035] Cave Sophie N., Wright Megan, von Stumm Sophie (2022). Change and stability in the association of parents’ education with children’s intelligence. Intelligence.

[B18-jintelligence-11-00035] Chan Chung-hong, Grill Christiane (2022). The Highs in Communication Research: Research Topics With High Supply, High Popularity, and High Prestige in High-Impact Journals. Communication Research.

[B19-jintelligence-11-00035] Childs Peter, Han Ji, Chen Liuqin, Jiang Pan, Wang Pan, Park Dongmyung, Yin Yuan, Dieckmann Elena, Vilanova Ignacio (2022). The Creativity Diamond—A Framework to Aid Creativity. Journal of Intelligence.

[B20-jintelligence-11-00035] Cole Eelizabeth R. (2009). Intersectionality and research in psychology. American Psychologist.

[B21-jintelligence-11-00035] Colom Roberto, Abad Francisco J., Quiroga M. Ángeles, Shih Pei C., Flores-Mendoza Carmen (2008). Working memory and intelligence are highly related constructs, but why?. Intelligence.

[B22-jintelligence-11-00035] Condon David M., Revelle William (2014). The international cognitive ability resource: Development and initial validation of a public-domain measure. Intelligence.

[B23-jintelligence-11-00035] Conway Andrew R. A., Cowan Nelson, Bunting Michael F., Therriault David J., Minkoff Scott R. B. (2002). A latent variable analysis of working memory capacity, short-term memory capacity, processing speed, and general fluid intelligence. Intelligence.

[B24-jintelligence-11-00035] Coyle Thomas R. (2022a). Processing speed mediates the development of tech tilt and academic tilt in adolescence. Intelligence.

[B25-jintelligence-11-00035] Coyle Thomas R. (2022b). Sex differences in spatial and mechanical tilt: Support for investment theories. Intelligence.

[B26-jintelligence-11-00035] Coyle Thomas R., Greiff Samuel (2021). The future of intelligence: The role of specific abilities. Intelligence.

[B27-jintelligence-11-00035] Cucina Jeffrey, Byle Kevin (2017). The Bifactor Model Fits Better Than the Higher-Order Model in More Than 90% of Comparisons for Mental Abilities Test Batteries. Journal of Intelligence.

[B28-jintelligence-11-00035] De Keersmaecker Jonas, Roets Arne (2017). ‘Fake news’: Incorrect, but hard to correct. The role of cognitive ability on the impact of false information on social impressions. Intelligence.

[B29-jintelligence-11-00035] Deary Ian J., Whalley Lawrence J., Lemmon Helen, Crawford John R., Starr John M. (2000). The Stability of Individual Differences in Mental Ability from Childhood to Old Age: Follow-up of the 1932 Scottish Mental Survey. Intelligence.

[B30-jintelligence-11-00035] Deary Ian J., Strand Steve, Smith Pauline, Fernandes Cres (2007). Intelligence and educational achievement. Intelligence.

[B31-jintelligence-11-00035] Demetriou Andreas, Mougi Antigoni, Spanoudis George, Makris Nicolaos (2022). Changing developmental priorities between executive functions, working memory, and reasoning in the formation of g from 6 to 12 years. Intelligence.

[B32-jintelligence-11-00035] Demetriou Andreas, Golino Hudson, Spanoudis George, Makris Nicolaos, Greiff Samuel (2021). The future of intelligence: The central meaning-making unit of intelligence in the mind, the brain, and artificial intelligence. Intelligence.

[B33-jintelligence-11-00035] Egghe Leo (2006). Theory and practise of the g-index. Scientometrics.

[B34-jintelligence-11-00035] Eid Michael, Krumm Stefan, Koch Tobias, Schulze Julian (2018). Bifactor models for predicting criteria by general and specific factors: Problems of nonidentifiability and alternative solutions. Journal of Intelligence.

[B35-jintelligence-11-00035] Erceg Nikola, Galić Zvonimir, Bubić Andreja (2022). Normative responding on cognitive bias tasks: Some evidence for a weak rationality factor that is mostly explained by numeracy and actively open-minded thinking. Intelligence.

[B36-jintelligence-11-00035] Ericsson Karl A. (2014). Why expert performance is special and cannot be extrapolated from studies of performance in the general population: A response to criticisms. Intelligence.

[B37-jintelligence-11-00035] Ericsson Karl A., Krampe Ralph T., Tesch-Römer Clemens (1993). The role of deliberate practice in the acquisition of expert performance. Psychological Review.

[B38-jintelligence-11-00035] Euler Matthew J., Schubert Anna-Lena (2021). Recent developments, current challenges, and future directions in electrophysiological approaches to studying intelligence. Intelligence.

[B39-jintelligence-11-00035] Fagan Joseph F., McGrath Susan K. (1981). Infant recognition memory and later intelligence. Intelligence.

[B40-jintelligence-11-00035] Feraco Tommaso, Cona Giorgia (2022). Differentiation of general and specific abilities in intelligence. A bifactor study of age and gender differentiation in 8- to 19-year-olds. Intelligence.

[B41-jintelligence-11-00035] Flynn James R. (1987). Massive IQ gains in 14 nations: What IQ tests really measure. Psychological Bulletin.

[B42-jintelligence-11-00035] Forthmann Boris, Förster Natalie, Souvignier Elmar (2022). Shaky Student Growth? A Comparison of Robust Bayesian Learning Progress Estimation Methods. Journal of Intelligence.

[B43-jintelligence-11-00035] Ganzach Yoav, Zisman Chen (2022). Achievement tests and the importance of intelligence and personality in predicting life outcomes. Intelligence.

[B44-jintelligence-11-00035] Gardner Howard E. (2011). Frames of Mind: The Theory of Multiple Intelligences.

[B45-jintelligence-11-00035] Geary David. C. (2022). Spatial ability as a distinct domain of human cognition: An evolutionary perspective. Intelligence.

[B46-jintelligence-11-00035] Gignac Gilles E. (2016). The higher-order model imposes a proportionality constraint: That is why the bifactor model tends to fit better. Intelligence.

[B47-jintelligence-11-00035] Gonthier Corentin, Grégoire Jacques (2022). Flynn effects are biased by differential item functioning over time: A test using overlapping items in Wechsler scales. Intelligence.

[B48-jintelligence-11-00035] Gustafsson Jan-Eric (1984). A unifying model for the structure of intellectual abilities. Intelligence.

[B49-jintelligence-11-00035] Haier Richard J. (2021). Are we thinking big enough about the road ahead? Overview of the special issue on the future of intelligence research. Intelligence.

[B50-jintelligence-11-00035] Haier Richard J., Siegel Benjamin V., Nuechterlein Keith H., Hazlett Erin, Wu Joseph. C., Paek Joanne, Browning Heather L., Buchsbaum Monte S. (1988). Cortical glucose metabolic rate correlates of abstract reasoning and attention studied with positron emission tomography. Intelligence.

[B51-jintelligence-11-00035] Hambrick David Z., Oswald Federick L., Altmann Erik M., Meinz Elizabeth J., Gobet Fernand, Campitelli Guillermo (2014). Deliberate practice: Is that all it takes to become an expert?. Intelligence.

[B52-jintelligence-11-00035] Harris Charles R., Millman K. Jarrod, van der Walt Stéfan J., Gommers Ralf, Virtanen Pauli, Cournapeau David, Wieser Eric, Taylor Julian, Berg Sebastian, Smith Nathaniel J. (2020). Array programming with NumPy. Nature.

[B53-jintelligence-11-00035] Hofer Gabriela, Mraulak Valentina, Grinschgl Sandra, Neubauer Aljoscha C. (2022). Less-Intelligent and Unaware? Accuracy and Dunning–Kruger Effects for Self-Estimates of Different Aspects of Intelligence. Journal of Intelligence.

[B54-jintelligence-11-00035] Hunt Earl (2010). Human Intelligence.

[B55-jintelligence-11-00035] Hunt Earl, Jaeggi Sussane M. (2013). Challenges for Research on Intelligence. Journal of Intelligence.

[B56-jintelligence-11-00035] Jauk Emanuel, Benedek Mathias, Dunst Beate, Neubauer Aljoscha C. (2013). The relationship between intelligence and creativity: New support for the threshold hypothesis by means of empirical breakpoint detection. Intelligence.

[B57-jintelligence-11-00035] Jensen Arthur R. (1998). The g Factor and the Design of Education. Intelligence, Instruction, and Assessment.

[B58-jintelligence-11-00035] Johnson Wendy, Nijenhuis Jan te, Bouchard Thomas J. (2008). Still just 1 g: Consistent results from five test batteries. Intelligence.

[B59-jintelligence-11-00035] Józsa Kriztian, Amukune Stephen, Zentai Gabriella, Barrett Karen C. (2022). School Readiness Test and Intelligence in Preschool as Predictors of Middle School Success: Result of an Eight-Year Longitudinal Study. Journal of Intelligence.

[B60-jintelligence-11-00035] Karama Sherif, Ad-Dab’bagh Yasser, Haier Richard J., Deary Ian J., Lyttelton Oliver C., Lepage Claude, Evans Alan C. (2009). Positive association between cognitive ability and cortical thickness in a representative US sample of healthy 6 to 18 year-olds. Intelligence.

[B61-jintelligence-11-00035] Karwowski Maciej, Dul Jan, Gralewski Jacek, Jauk Emanuel, Jankowska Dorota M., Gajda Aleksandra, Chruszczewski Michael H., Benedek Mathias (2016). Is creativity without intelligence possible? A Necessary Condition Analysis. Intelligence.

[B62-jintelligence-11-00035] Koch Marco, Becker Nicolas, Spinath Frank M., Greiff Samuel (2021). Assessing intelligence without intelligence tests. Future perspectives. Intelligence.

[B63-jintelligence-11-00035] Koenig Katherine A., Frey Mederith C., Detterman Douglas K. (2008). ACT and general cognitive ability. Intelligence.

[B64-jintelligence-11-00035] Kyllonen Patrick C., Zu Jiyun (2016). Use of response time for measuring cognitive ability. Journal of Intelligence.

[B65-jintelligence-11-00035] Kyllonen Patrick C., Christal Raymond E. (1990). Reasoning ability is (little more than) working-memory capacity?. Intelligence.

[B66-jintelligence-11-00035] Laureys Felien, Waelle Silke De, Barendse Maria T., Lenoir Matthiew, Deconinck Federick J. A. (2022). The factor structure of executive function in childhood and adolescence. Intelligence.

[B67-jintelligence-11-00035] Liu Zhigao, Yin Yimei, Liu Weidong, Dunford Michael (2015). Visualizing the intellectual structure and evolution of innovation systems research: A bibliometric analysis. Scientometrics.

[B68-jintelligence-11-00035] Lynn Richard, Meisenberg Gerhard (2010). National IQs calculated and validated for 108 nations. Intelligence.

[B69-jintelligence-11-00035] Lynn Richard, Harvey John, Nyborg Helmuth (2009). Average intelligence predicts atheism rates across 137 nations. Intelligence.

[B70-jintelligence-11-00035] Marrone Rebecca, Taddeo Victoria, Hill Gillian (2022). Creativity and Artificial Intelligence—A Student Perspective. Journal of Intelligence.

[B71-jintelligence-11-00035] Massie Marie-Helene, Puozzo Isabelle Capron, Boutet Marc (2022). Teacher Creativity: When Professional Coherence Supports Beautiful Risks. Journal of Intelligence.

[B72-jintelligence-11-00035] Mayer John D., Salovey Peter (1993). The intelligence of emotional intelligence. Intelligence.

[B73-jintelligence-11-00035] Mayer John D., Caruso David R., Salovey Peter (1999). Emotional intelligence meets traditional standards for an intelligence. Intelligence.

[B74-jintelligence-11-00035] McGrew Kevin S. (2009). CHC theory and the human cognitive abilities project: Standing on the shoulders of the giants of psychometric intelligence research. Intelligence.

[B75-jintelligence-11-00035] Morgan Grant, Hodge Kari, Wells Kevin, Watkins Marley (2015). Are Fit Indices Biased in Favor of Bi-Factor Models in Cognitive Ability Research?: A Comparison of Fit in Correlated Factors, Higher-Order, and Bi-Factor Models via Monte Carlo Simulations. Journal of Intelligence.

[B76-jintelligence-11-00035] Neubauer Aljoscha C. (2021). The future of intelligence research in the coming age of artificial intelligence—With a special consideration of the philosophical movements of trans- and posthumanism. Intelligence.

[B77-jintelligence-11-00035] Novikova Irina A., Gridunova Marina V., Novikov Alexey L., Shlyakhta Dmitriy A. (2022). Cognitive Abilities and Academic Achievement as Intercultural Competence Predictors in Russian School Students. Journal of Intelligence.

[B78-jintelligence-11-00035] Nusbaum Emily C., Silvia Paul J. (2011). Are intelligence and creativity really so different?: Fluid intelligence, executive processes, and strategy use in divergent thinking. Intelligence.

[B79-jintelligence-11-00035] Oberauer Klaus, Süβ Heinz-Martin, Wilhelm Oliver, Wittmann Werner W. (2008). Which working memory functions predict intelligence?. Intelligence.

[B80-jintelligence-11-00035] Otero Inmaculada, Salgado Jesús F., Moscoso Silvia (2022). Cognitive reflection, cognitive intelligence, and cognitive abilities: A meta-analysis. Intelligence.

[B81-jintelligence-11-00035] Panesi Sabrina, Bandettini Alessia, Traverso Laura, Morra Sergio (2022). On the Relation between the Development of Working Memory Updating and Working Memory Capacity in Preschoolers. Journal of Intelligence.

[B82-jintelligence-11-00035] Pásztor Attila, Magyar Andrea, Pásztor-Kovács Anita, Rausch Aattila (2022). Online Assessment and Game-Based Development of Inductive Reasoning. Journal of Intelligence.

[B83-jintelligence-11-00035] Pesta Bryan, John Fuerst, Kirkegaard Emil O. W. (2018). Bibliometric Keyword Analysis across Seventeen Years (2000–2016) of Intelligence Articles. Journal of Intelligence.

[B84-jintelligence-11-00035] Pesta Bryan J. (2018). Bibliometric analysis across eight years 2008–2015 of Intelligence articles: An updating of Wicherts (2009). Intelligence.

[B85-jintelligence-11-00035] Rad Mostafa S., Martingano Alison J., Ginges Jeremy (2018). Toward a psychology of Homo sapiens: Making psychological science more representative of the human population. Proceedings of the National Academy of Sciences.

[B86-jintelligence-11-00035] Rammstedt Beatrice, Lechner Clemens M., Danner Daniel (2018). Relationships between personality and cognitive ability: A facet-level analysis. Journal of Intelligence.

[B87-jintelligence-11-00035] Rosas Ricardo, Espinoza Victoria, Martínez Camila, Santa-Cruz Catalina (2022). Playful Testing of Executive Functions with Yellow-Red: Tablet-Based Battery for Children between 6 and 11. Journal of Intelligence.

[B88-jintelligence-11-00035] Roth Bettina, Becker Nicolas, Romeyke Sara, Schäfer Sarah, Domnick Florian, Spinath Frank M. (2015). Intelligence and school grades: A meta-analysis. Intelligence.

[B89-jintelligence-11-00035] Rusche Marianna M., Ziegler Matthias (2022). The interplay between domain-specific knowledge and selected investment traits across the life span. Intelligence.

[B90-jintelligence-11-00035] Sideridis Georgios, Alahmadi Maisa (2022). Estimation of Person Ability under Rapid and Effortful Responding. Journal of Intelligence.

[B91-jintelligence-11-00035] Spearman Charles (1927). The Measurement of Intelligence. Nature.

[B92-jintelligence-11-00035] Sternberg Robert J. (1985). Beyond IQ: A Triarchic Theory of Human Intelligence.

[B93-jintelligence-11-00035] Sternberg Robert J. (2019). A theory of adaptive intelligence and its relation to general intelligence. Journal of Intelligence.

[B94-jintelligence-11-00035] Sternberg Robert J. (2022). The Search for the Elusive Basic Processes Underlying Human Intelligence: Historical and Contemporary Perspectives. Journal of Intelligence.

[B95-jintelligence-11-00035] Sternberg Robert J., Siriner I., Oh J., Wong C. H. (2022). Cultural Intelligence: What Is It and How Can It Effectively Be Measured?. Journal of Intelligence.

[B96-jintelligence-11-00035] Suh Woong, Ahn Seongjin (2022). Utilizing the Metaverse for Learner-Centered Constructivist Education in the Post-Pandemic Era: An Analysis of Elementary School Students. Journal of Intelligence.

[B97-jintelligence-11-00035] Tatel Corey E., Tidler Zachary R., Ackerman Phillip L. (2022). Process differences as a function of test modifications: Construct validity of Raven’s advanced progressive matrices under standard, abbreviated and/or speeded conditions—A meta-analysis. Intelligence.

[B98-jintelligence-11-00035] Tourva Anna, Spanoudis George (2020). Speed of processing, control of processing, working memory and crystallized and fluid intelligence: Evidence for a developmental cascade. Intelligence.

[B99-jintelligence-11-00035] van der Maas Han L. J., Kan Kees-Jan, Borsboom Denny (2014). Intelligence is what the intelligence test measures. Seriously. Journal of Intelligence.

[B100-jintelligence-11-00035] van der Maas Han L. J., Snoek Lukas, Stevenson Claire E. (2021). How much intelligence is there in artificial intelligence? A 2020 update. Intelligence.

[B101-jintelligence-11-00035] Van Der Maas Han, Kan Kees-Jan, Marsman Maarten, Stevenson Claire E. (2017). Network Models for Cognitive Development and Intelligence. Journal of Intelligence.

[B102-jintelligence-11-00035] Van Eck Nees J., Waltman Ludo (2007). Bibliometric mapping of the computational intelligence field. International Journal of Uncertainty, Fuzziness and Knowledge-Based Systems.

[B103-jintelligence-11-00035] von Stumm Sophie, Plomin Robert (2015). Socioeconomic status and the growth of intelligence from infancy through adolescence. Intelligence.

[B104-jintelligence-11-00035] Wai J. (2020). Communicating intelligence research. Journal of Intelligence.

[B105-jintelligence-11-00035] Wai Jonathan, Worrell Frank C. (2021). The future of intelligence research and gifted education. Intelligence.

[B106-jintelligence-11-00035] Wai Jonathan, Lee Matthew H., Kell Harrison J. (2022). Distributions of academic math-verbal tilt and overall academic skill of students specializing in different fields: A study of 1.6 million graduate record examination test takers. Intelligence.

[B107-jintelligence-11-00035] Wicherts Jelte M. (2009). The impact of papers published in Intelligence 1977–2007 and an overview of the citation classics. Intelligence.

[B108-jintelligence-11-00035] Wilhelm Oliver, Kyllonen Patrick (2021). To predict the future, consider the past: Revisiting Carroll (1993) as a guide to the future of intelligence research. Intelligence.

[B109-jintelligence-11-00035] Willerman Lee, Schultz Robert, Rutledge J. Neal, Bigler Erin D. (1991). In vivo brain size and intelligence. Intelligence.

[B110-jintelligence-11-00035] Zisman Chen, Ganzach Yoav (2022). The claim that personality is more important than intelligence in predicting important life outcomes has been greatly exaggerated. Intelligence.

